# Twelve things we have learned from medical students during the Pandemic

**DOI:** 10.15694/mep.2021.000077.1

**Published:** 2021-03-25

**Authors:** Julian Pecora, Tarun Sen Gupta, Daniel Zou, Kathy Kwan, Hannah Matthews, Steve Trumble

**Affiliations:** 1 James Cook University Medical Students’ Association; 2 James Cook University; 3 The Australian Medical Students’ Association and the University of Melbourne; 4 The University of Queensland Medical Society; 5 Medical Students’ Council of Western Australia and the University of Western Australia; 6 The University of Melbourne

**Keywords:** Medical education, COVID-19, innovation, students

## Abstract

This article was migrated. The article was marked as recommended.

While the COVID-19 pandemic has disrupted every aspect of modern life, including medical education, the response has been remarkable. Ingenuity and innovation have flourished in the face of adversity. An international community of practice has blossomed in response to the challenges posed by COVID-19. Communication and sharing of information have been a hallmark of this community.

In Australasia, the Medical Education Collaborative Committee hosted a series of meetings and webinars which enabled educators from all Australian and New Zealand medical schools to share experiences, solutions and resources. This group is auspiced by the Medical Deans Australia and New Zealand, which is the peak body representing professional entry-level medical education, training and research.

One of these webinars focused on the student experience, featuring a panel of Australasian medical students drawn from a range of medical schools. The discussion during this session was wide reaching, including topics such as communication, co-designing curriculum changes, the importance of compassion, innovative practice, and meaningful student participation in placements. The ideas drawn from the panel discussion augmented by rich audience participation form the basis of the twelve tips presented in this paper. While derived from the experience in Australia and New Zealand, the authors believe these lessons are likely to be relevant in other jurisdictions.

## Introduction

Every aspect of modern life, in almost every corner of the globe has been disrupted by the COVID-19 pandemic. Hays *et al*. observe that health professional education is no exception, being situated within the healthcare system, universities, and the broader community ( Hays *et al.*, 2020). Rose notes the disruption of medical education requires ‘intense and prompt attention from medical educators’ ( Rose, 2020).

Health professional educators have indeed responded to the most severe global disruption since the Second World War, with an emerging literature describing shared experiences and similar responses. The online medical education journal MedEdPublish described a total of 39 papers published in the first three months of the pandemic, addressing topics such as curriculum adaptation, guidelines for using technology, assessment adaptation, impact on students, faculty and career development, and conference adaptation ( Hays *et al.*, 2020). These authors noted a wide range of participants contributed to this literature, including those in leadership roles, teachers, examiners and learners.

All Australasian medical schools were affected by COVID-19. One national response saw the Medical Education Collaborative Committee hosting a series of weekly meetings which enabled educators from all Australian and New Zealand medical schools to share experiences, solutions and resources. This group is auspiced by the Medical Deans Australia and New Zealand (MDANZ), the peak body representing professional entry-level medical education, training and research in Australia and New Zealand and comprising the Deans of the 23 medical schools in the two countries ( Medical Deans Australia and New Zealand, 2020a).

Medical Deans Australia and New Zealand led some important policy development through the pandemic, releasing statements on medical students’ contribution to the health workforce response to COVID-19 and the preparedness of medical schools to boost hospital capacity with new Assistant in Medicine (AIM) roles ( Medical Deans Australia and New Zealand, 2020b), as well as supporting all medical students to train in mental health first aid ( Medical Deans Australia and New Zealand, 2020c). A series of webinars was also held across Australia and New Zealand looking at issues such as curricular responses to COVID-19, wellbeing of staff and students, leadership and e-portfolios.

One of these webinars, held in June 2020, focused on the student experience. Entitled ‘AC/DC (After COVID / During COVID) - TNT (Try New Tricks)’, this webinar featured a panel of Australasian medical students drawn from a range of medical schools, an international medical student voice and also the Presidents of both the Australian and the New Zealand Medical Students’ Associations, with facilitation by two academics. The webinar was supported by Medical Deans Australia and New Zealand with over 120 educators and medical students registering to attend.

The webinar aimed to allow current and future medical educators to gain a better understanding of medical students’ experiences during the COVID-19 pandemic and what they expect their world to be like afterwards. During the session, the panel responded to audience questions and provided insights into the experience of Australasian medical students as:

• A clinical learner - What has been their experience of teaching and learning during the pandemic and what are the lessons for the future?
• A developing professional - What has been the impact of the virus and what recommendations are there for ‘returning to clinic’?
• A person, peer and friend - What is our role and the students’ role in providing peer support and teaching, academic support, and emotional support?

These twelve tips are based on the lively discussion during the forum between students and medical educators from across Australia and New Zealand, and may be relevant to other jurisdictions. A striking feature was the many rich insights from student participants. In the words of the Talmud, ‘I have learned much wisdom from my teacher, more from my colleagues and the most from my students’ ( Etshalom, nd).

## Tip 1: Clear and consistent communication is critical

Clear communication is essential in a crisis. Students - and other stakeholders - need consistent, honest and timely messages. Our experience demonstrates that students accept uncertainty, particularly in rapidly changing times, and appreciate understanding what is known and what isn’t. We noted record attendance and engagement at COVID-19 update webinars and other educational sessions - no ‘sign-in sheet’ was needed. Why? Students wanted to hear for themselves the messages which were contemporary, relevant and important. Perhaps this is a lesson for our other educational offerings.

Communications need to cover many elements. Students were obviously invested in how COVID-19 impacted on their studies - as well as many other things. How are other years affected? What is the impact on the health system - and their future? Students were also interested in general information on COVID-19, the local scene, and wider issues and appreciated the input of staff members with special expertise in this area.

The format of these sessions is also important. Face-to-face meetings (where permissible and feasible) and the increasingly common webinars allowed real time discussion and interaction, and could be recorded for future reference. The ‘chat’ function in webinars was widely used and was found to be efficient (similar questions could be grouped; sometimes students answered each other’s questions or shared resources) and allowed for respectful engagement. Such live sessions were often followed up with written messages which allowed the key messages to be summarised and clearly documented. Specific communications from medical schools had to be balanced against university-wide and community-wide messages and general public health measures. We rapidly understood the need to guard against message-fatigue, and found ways to curate the multiple communications so they were readily available for future reference.

Consider also the stakeholders. Students were key - but others need to be in the loop, including staff, adjuncts, clinicians and the health care managers. Consider carefully who needs to know what and when. We noted sometimes we got this wrong. For example, on at least one occasion messages about timetable changes went out to students before staff were aware. Beware, also, of inadvertent messaging, for example one university became aware that invoices were being sent to international students at the height of the uncertain period.

## Tip 2: Co-design curriculum changes with students

Students are experts in the curriculum, and therefore ideally placed to advise on changes. If asked they will generally be thoughtful and generous with their opinions, providing constructive feedback. A shared adversary - the pandemic - can lead to shared determination and shared resources. Students understand we are all facing the same question: how do we get through this together with finite (limited) resources? Engaging students in the co-design can enable creative and ingenious solutions. We found students can be surprisingly adaptable when they feel they are part of the solution.

Senior students, who have just been through the course, have special expertise. They know the curriculum, the students and the environment, and will often identify innovative solutions. For example, their first-hand knowledge of how things actually work on the ground can be invaluable in redesigning clinical rosters.

One webinar participant from Flinders University noted students are very much aware of their future responsibilities as interns or doctors and are not necessarily just interested in passing examinations when they are not ready to do so. Students are very creative in designing assessment approaches that will work. All educators will recognise they are also adept at circumventing assessment approaches that do not work. They are also very creative in circumventing remote proctoring, so their advice in designing COVID-appropriate assessment was very helpful. We also found the students to be very constructive conversation partners.

And, unsurprisingly, if solutions are co-designed with students they are more likely to be accepted. There was also an observation that it is a valuable experience for students to finally have some control and agency over their own assessment. In most programs, assessment is purely done TO the students and not WITH them. However, once they graduate they are expected to take agency in their own assessment processes, although we have never prepared them for it (L Schuwirth, 2020 personal communication, 14 July).

## Tip 3: Forge genuine partnerships with student leaders

The importance of engagement and genuine partnership with leaders of the student body cannot be overstated (the nature and structure of medical student leadership groups in Australia are presented in *Tip 6*). Medical student leaders are elected by their peers, in part to provide representation and advocacy on any emerging issues affecting the curriculum or student experience. As the COVID-19 pandemic could be considered the greatest of such issues in recent history, student leaders were busier than ever before.

Staff consultation with student leadership teams took on several different formats. Much communication occurred formally via recurring, pre-scheduled meetings in which capturing an ever-elusive status quo was the objective. Some consultation was urgent and ad-hoc as the situation necessitated, such as with news of imminent border closures. Unanticipated evening phone calls between both parties were not an unusual occurrence. Student leaders were a vital part of the change management process, being able to provide guidance not just on the ‘what’ - changes to curricular content - but also the ‘how’ - the best way to introduce changes and sell the message. The student leadership team was often proactive in surveying, synthesising and triaging their colleagues’ concerns, as well as identifying weaknesses in any proposed changes. Suggested changes that may seem minor to academic staff (such as the timing of recess weeks or rescheduling assessments) may be perceived as an enormous inconvenience by students and vice versa, even more so in the heightened tensions of an unprecedented pandemic. Encouragingly, despite pervasive uncertainty, levels of satisfaction and confidence among the general student body seemed buoyed by the strength of the partnership between student leaders and staff.

Developing a robust and transparent relationship with student leaders is particularly beneficial as they are often the first ‘port of call’ for their colleagues in need. Whether for status updates or disclosure of serious matters, medical students commonly reach out to trusted peer representatives before official university channels, owing to a perception of easier accessibility or confidentiality concerns. Social media networks between peers mean that student leaders are never ‘out-of-office’ and replies can typically be expected within minutes. Clearly, both university staff and student leaders share the goal of looking out for the student body and thus a genuine, strong partnership between the two parties is valuable and logical.

## Tip 4: A bit of humanity and compassion goes a long way. We should all practise kindness

Humanity and compassion are core values in promoting patient wellbeing in the medical profession, so would we be remiss not to address it in medical education? The importance of translating compassion into the pedagogy of medicine cannot be understated. To combat the spread of COVID-19, restrictions were implemented that drastically changed the landscape and functioning of day-to-day life. The resultant isolation and restrictions impacted medical students and educators in many capacities. Each of the rapidly evolving challenges faced by students and faculty required some understanding and compassion to work through together. We noted students struggling to engage with material taught in a considerably different format and environment than what they were previously accustomed to. Similarly, educators and staff faced unforeseen challenges of working from home. Most of us needed time to adjust, and that is okay.

What did we learn? We learned the importance of listening to student representatives who bring forth the concerns of the student body, and really listen. We worked to overcome compassion fatigue. We realised students are more understanding of the implemented changes when it has been communicated effectively and when they feel as though they have been truly heard.

We learned to connect regularly in order to add doses of humanity. Many programs elicited great student interest and engagement in real-time virtual opt-in mindfulness sessions with student support teams. Likewise, efforts to discuss mental health or provide check-ins during tutorials with educators were appreciated by students. Additionally, providing feedback through regular surveys or open fora allowed students to not only convey their constructive criticisms, but to also show their appreciation for educators and staff who went above and beyond to ensure a smooth transition during COVID-19. Effective two-way communication allows all parties to gain insight into one another’s perspectives to humanise the problems at hand.

We recognised that we are all human. It is easy to briefly lose sight of this and turn to the most efficient blanket methods in solving problems at an organisational level. However, a little dose of humanity goes a long way. We understood that these are not just cohorts of medical students, but individual diligent students with real struggles; and these are not just medical faculties, but individual passionate educators and staff working with best interests in mind. Humanity and compassion should be intertwined in efforts of current and future planning. Ultimately, whether educators or students, we should all practise kindness.

In the words of the Dalai Lama, ‘If you want others to be happy, practice compassion. If you want to be happy, practice compassion’ ( Dalai Lama XIV, 1998).

## Tip 5: Students are not a homogenous group. Get to know them and understand their individual needs

A major impact of COVID-19 that has been widely acknowledged is how it accentuates and magnifies current inequalities and system deficiencies. The pandemic has exposed cracks in institutional support systems and government programs, oftentimes revealing the truly vulnerable and precarious situation of some members of society. Medical schools are no different. As government, hospital and university systems start to crack, everyone feels exposed, particularly those who are most vulnerable.

Through COVID-19, numerous medical student concerns have risen to the fore; many of these unknown and unconsidered by the support systems and structures instituted by universities or hospitals. Many of these concerns are relevant to all medical students, such as medical student safety and wellbeing, and interruptions in medical education. Other concerns pertain to particular student subgroups where additional support can and should be provided. What COVID-19 has done is help provide greater insight and definition to the various smaller subgroups of medical students which once seemed to all fit into a homogenous collective.

Some of the student subgroups that have a demonstrable need during COVID-19 and who can and have benefitted from additional university or government support include:

• International medical students
• Medical students from interstate and rural/regional locations
• Medical students with dependents
• Medical students with disabilities or medical conditions
• Aboriginal and Torres Strait Islander medical students
• Medical students who identify as queer or gender non-binary

But it is also important to move beyond these superficial and potentially static labels categorising student needs and concerns. What underlies the principle of identifying unique student subgroups that can be supported is a broader cognisance and deeper understanding of the demographics outlining one’s own medical student cohort. To be able to tailor support to students, an intimate understanding of one’s cohort at hand is essential.

As described in *Tip 3*, the role of student leadership is vital in this process. They have played a major role in engaging with membership to identify student concerns and to enable institutional support to be matched with student needs. Simultaneously, there is an immense role for universities and hospitals to play. Due to the potential of sharing highly sensitive information, it is incumbent on hospitals and medical schools to transparently identify the processes and procedures that will help protect student privacy and confidentiality; and for hospitals and medical schools to proactively discuss what potential support students can receive if they were to identify particular concerns. Successful student support is a joint effort between medical schools and medical students.

Alarmingly, without COVID-19 exposing the needs of these specific student subgroups, these students would have continued to make do and struggle through medical school. This stress can pervade and underscore a student’s entire medical school journey, and contribute to poor mental health and potentially physical health outcomes. Moreover, it is disappointing that this additional burden is often considered the ‘price to pay’, ‘builds character resilience’ or the ‘show of commitment’ that one is required to complete medical school and can go completely unrecognised as issues that universities or hospitals may provide support for.

Ultimately, medical students are an extremely diverse group of individuals. Beyond COVID-19 and 2020, medical schools will continue to attract a wildly diverse cohort of students. It is therefore increasingly important to acknowledge and respond to this student diversity and not mistake medical students as a homogenous group.

## Tip 6: Utilise student channels and networks

In Australasia, medical students enjoy comprehensive, multi-level representation and governance networks, managed by their peers. Each medical school has a local student-run society - colloquially termed the ‘MedSoc’ - which provides advocacy for the student body, organises academic and social events, sponsors student activities and overall aims to enhance the student journey. One example is the James Cook University Medical Students’ Association (JCUMSA), of which author JP is President. Most Australian states also have a medical student council that further represents students and MedSocs on state-wide issues, such as internship allocations. For instance, author HM is Chair of the Medical Student Council of Western Australia (MSCWA). Connecting all 17,000 students nationally, as well as their MedSocs and state councils, is a peak representative body known as the Australian Medical Students’ Association (AMSA), of which author DZ is President. Medical student leaders meet routinely across a range of fora for the purpose of collective advocacy, policy formation, troubleshooting and professional development.

At the onset of the pandemic in Australia, the initial responses of our 21 medical schools differed markedly. Some institutions elected to adopt an immediate ‘pause’ to clinical placements across all year levels, some encouraged students to continue their attachments where safe, and others worked with local and state-based regulatory bodies in pioneering a student surge workforce. Examinations were postponed at some sites, shifted online for others, or transformed entirely in format (e.g. open-book or unproctored). By late March, it became apparent that the experience of students in 2020 would vary considerably between the different schools and, in some cases, even between campuses of the same university. While no one could be certain which decisions were best suited to the uncharted times ahead, it seemed prudent to share intelligence and compare and monitor the contrasting approaches being endorsed by different providers.

Owing to the high transparency, connectedness and collegiality of the aforementioned student representative bodies across Australia, seemingly every status update (spanning assessment, quarantine leave policies, fee reduction, etc.) was openly and rapidly shared between these vast student networks. One such channel was an iterative, live document in which MedSoc Presidents collaboratively tabulated these changes, making them instantly accessible across the country. Medical student leaders interact constantly, and never more than in 2020, a year which has demanded large-scale problem-solving and in which critical negotiations with stakeholders such as state health departments have been commonplace. These factors afford student leaders an immense knowledge of movements in medical education across the country. Some MedSocs even recall being approached by their College faculties, who were desperately seeking clarity as to what rules and protocols other providers had implemented! While staff absolutely have their own networks, student ones appear to be more active and their information more current. Providers should recognise the medical student network as a rich, underutilised and powerful information source, and one that circumvents the bureaucratic processes which may impede discussions between schools.

## Tip 7: Focus on what is essential

A pervasive question posed to us all during the COVID-19 pandemic has been ‘what is essential?’ Is your travel essential? Are you an essential worker? Are you gathering for an essential reason? Unsurprisingly, as we continually structure our daily activities around this theme of essentiality, we suggest it must colour the design of medical education in the post-COVID-19 era. For some time, there has been a decline in student engagement with traditional teaching methods across medical curricula. Educators have noted diminishing attendance at conventional didactic sessions, as students increasingly value the convenience and digestibility of online materials ( Emanuel, 2020). Traditional lecture and tutorial formats with enforced attendance constitute a rigid ‘one-size-fits-all’ approach for ever-diverse medical student cohorts with heterogeneous needs. In some respects, COVID-19 has functioned as a much-needed initial catalyst for a necessary paradigm shift in medical education, where flexibility and competency attainment are emphasised rather than lectures attended or hours accrued. In the same way that contemporary teaching espouses care that is patient-centred, so too should we call for medical education that is proudly student-centred.

By definition, arriving at what is essential also means re-evaluating the extraneous. Poor mental health and burnout phenomena have been characterised more extensively in medical students than perhaps any other subset of the population. The contributing factors are broad, complex and vary by context, though a paucity of time for leisure, physical activity and for family encounters are some cited extra-institutional factors, which may be common between schools ( Boni *et al*., 2018). As the pandemic obliges educators to streamline their curricula, we encourage them to consider if it is worth ever fully returning to that which was deemed unnecessary. Clearly, leaving behind the non-essential can only afford students more time for the self-care and extracurricular pursuits instrumental to their wellbeing.

In 1978, Abrahamson described a number of diseases of the curriculum that are still relevant today ( Abrahamson, 1978). These include ‘curriculosclerosis’, or ‘hardening of the categories’ which he notes is, ‘by far the most crippling disease, and tragically also one of the most prevalent’. His second major disease entity, ‘carci-noma of the curriculum’, is characterised by ‘seemingly uncontrolla-ble growth of one segment or component of the curriculum’. COVID-19 has allowed us to compress years of change into weeks and critically evaluate what curricular components have become hardened and/or uncontrollable. Let us continue to investigate our curricula, to diagnose and treat it and to formulate management plans in consultation with our students and our colleagues. If treatment is radical, perhaps involving extensive surgery, then so be it, as the price we pay for good health.

The focus on what is core means we can ask the question of every piece of curricular content: is this essential, useful, or bonus? While formal evaluations are pending, early feedback is that many students have been able to meet all of their essential learning outcomes amidst the pandemic, and many of their useful and bonus ones.

COVID-19 has therefore forced us to think about other ways of meeting learning outcomes, with alternative formats and resources. Many questions arise: for example, what *has* to be done face-to-face? Flexibility may suit some groups of students, as outlined in *Tip 5.* Many of these solutions involve creative uses of Information Technology, described in the following *Tip.* A robust blueprinting process has enabled educators to map their COVID curriculum to the pre-COVID one, and may form a template for the new normal.

## Tip 8: Information Technology is your friend. Make friends with your IT team

Many of the innovative solutions to the challenges posed by COVID-19 involved creative use of Information Technology (IT). Multiple platforms have been used for teaching and assessment, depending on factors such as scale, cost, technical requirements, reliability, security features and available technical support. The Australian College of Rural and Remote Medicine identified a number of advantages of the Zoom™ platform for use in their online examinations: it was web-based (therefore there was no software to download) and used substantially less bandwidth, which is important in rural and remote locations ( Lewandowski *et al*., 2020).

Educators and students rapidly became familiar with the various platforms used for telecommunications. Videoconferencing connectivity was checked, and etiquette established, including the use of the mute function, appropriate backgrounds, and suitable dress. The leading contender for the phrase of the year must surely be, ‘You’re on mute.’ Participants rapidly made use of enhanced functionality such as the chat function - which in many cases was noted to improve communication and interaction. We learned to share screens, use discussion rooms and conduct online polls. Most importantly we realised that in every group there was usually one person who could overcome most IT problems - and that person was usually a student.

Some programs redesigned their curriculum to take advantage of the opportunities offered by IT. For example, at James Cook University, two of the traditional 6-week rotations were replaced at the height of the pandemic by a Structured Clinical Online Learning (SCOL) term. Each SCOL aimed to compress the lectures, tutorials and other theoretical teaching delivered over a 6-week period into 3 weeks. The clinical component was delivered later in the year when students were permitted to return to the clinical setting, as a complementary series of 3-week Intensive Clinical Placements.

While designing the SCOL terms took considerable effort from the academic staff (co-designing with the students as in *Tip 2*), in many ways the success of this approach depended on the efforts of the professional and technical team working behind the scenes. Rosters had to be devised, teaching sessions planned, and academics booked to attend as needed. This was a major project, with logistics managed by the professional team leaders who had to maintain clear communications with over 180 students across three clinical school sites, 100-plus teaching staff and many others involved in administration and support. Over 300 separate Zoom™ meetings were run over 6 weeks, with group sizes from 12 - 180. Critical to the success of this approach was the full-time presence of a dedicated technical assistant who was available to set up meetings, to problem-solve and to trouble-shoot.

One example of case-based teaching in the General Practice rotation illustrated the benefits of this approach. A tutorial was constructed around a real patient with an evolving problem, who had consented to take part. The patient attended their regular clinic to see their usual General Practitioner, who was also a staff member, and were linked by Zoom™ to the student group. As in other forms of problem based learning, the students had spent some time exploring the case before meeting the patient, and identifying the main problems from the available information. They then discussed aspects of the case with the patient, and formulated possible diagnoses and a management plan.

Evaluations suggested this format met the objectives of exposing students to real clinical problems, and making explicit the clinical reasoning process. In addition, this approach seemed to work well for the patient (i.e. attending their regular clinic to see students via videoconference was less threatening than speaking to a group in a tutorial room) and promoted more interactivity amongst the students who used the chat function extensively to ask questions and suggest possible diagnoses or areas to explore.

## Tip 9: Continuing meaningful student participation in placements

While medical students absolutely require clinical placements for experiential learning and skills development, the healthcare setting becomes an increasingly dangerous place in the context of a novel pandemic. There is a precarious balance: on one hand is the perceived and real duty of care as an education provider; on the other is the need to supply next year’s intake of work-ready graduates to a workforce already under strain. While a pandemic may be seen as a unique and rewarding setting for learning, would this optimism remain if students became critically unwell or died?

In Australia, the solution which emerged took the form of optional enlistment for senior medical students into a surge workforce, primarily via a purpose-built position termed the Assistant in Medicine (AIM) ( Monjur, 2020). Although early AIM concept design occurred on a national scale, specific terms and conditions (e.g. scope of practice, award rate, working hours) were generally decided by state-based health systems or even local Hospital and Health Services. As AIMs, students could therefore attain key competencies and maintain patient contact while performing work as integrated, employed members of the healthcare team. Under supervision, AIMs were able to perform many duties of the junior doctor, including clinical note-taking, attending to ward calls and various minor procedures such as peripheral intravenous cannulation ( Monjur, 2020). Final-year students who elected not to opt-in to the AIM program would continue clinical placements in a more conventional sense.

One factor that significantly bolstered student approval of the AIM concept was the swift recommendation of key governing principles for student employment by entities such as MDANZ ( Medical Deans Australia and New Zealand, 2020d). It was evident that such principles had been created with thorough consultation and a careful, measured consideration of potential student concerns. For instance, safety in the form of adequate access to personal protective equipment, indemnity, supervision and remuneration were among the recommendations. At one university, an early expression of interest survey amongst final-year medical students indicated that approximately 90% of the cohort would enrol for the AIM scheme. Many expressed feeling an inherent sense of duty to assist however possible.

Although the AIM approach brought many advantages, it was no panacea. What would be done for students who were unable to feasibly work or continue placement, such as medical students with dependents now at home given school closures, or for students who are immunocompromised? What was the difference between final-year students already expected to occupy a ‘sub-intern’ role in their particular program through contribution to patient care and the new AIM role (and why does only the latter get paid for similar work?) How could we be certain that students who elected not to opt-in for whatever reason would not be disadvantaged with respect to career prospects, supposing certain specialty colleges award points for or look favourably on AIMs? Is it really then an ‘opt-in’ system when not doing so could come at a cost? While answers to all of the above may not yet be forthcoming, they are worth contemplating in the case of subsequent waves or future crises.

In the end, formal recruitment and deployment of AIMs ultimately only materialised in those states which were hardest hit by the COVID-19 crisis. Irrespective, the substantial contribution that senior medical students can make to the healthcare system should be considered in future planning.

## Tip 10: Promote near peer teaching and support

In its initial stages, the rapid onset and uncertain forecast of the pandemic required educators to make swift decisions about every aspect of the teaching experience. How could student and staff safety be prioritised and effectively balanced with the need for continued, quality learning? One method employed with considerable success at James Cook University, Queensland, was a novel online near-peer teaching program formed in response to COVID-19. Near-peer teaching (NPT) is a well-established pedagogical format in Australasia and internationally which sees students adopt educator roles to teach their fellow junior peers (de Menezes and Premnath, 2016; Khaw and Raw, 2016). At James Cook University, NPT had been utilised in various forms but it proved especially valuable while the pandemic necessitated continued interruptions to in-person classes and clinical placements. Co-championed and co-designed by a final (6th) year student and members of faculty, our NPT format saw over 60 volunteer final year students deliver focussed specialty teaching to their near-peers in the penultimate cohort. The senior students collaborated in pairs to provide weekly videoconferenced sessions to groups of six junior students across rotations in obstetrics and gynaecology, mental health, general practice, surgery and internal medicine. Learning outcomes were jointly developed by the student teachers and program creators and emphasised clinical, ‘on-the-wards’’ knowledge that could be missed without access to conventional hospital placements during COVID-19.

Student recipients (near-peer-learners, NPLs) of the NPT program generally reported high levels of satisfaction with this teaching method. A commonly cited basis for this satisfaction was the perceived ‘approachability’ of the student teachers. Compared to sessions with usual educators (typically senior registrars or consultants), many NPLs found themselves less apprehensive about asking questions and better able to engage with the content during teaching. This was reportedly due to (1) a narrower knowledge and authority gap between teacher and student, such that NPLs did not feel ‘judged’ for ineptitude; (2) that the volunteer student teachers had no formal role in assessment of the NPLs and (3) the content was highly-tailored to the NPLs’ specific learning needs for the rotation, which is sometimes a challenge for hospital-based clinicians who are invited to teach. Ultimately, we found NPT to be a resource-light, effective method for continued teaching of penultimate year medical students during the COVID-19 pandemic. In the event of subsequent waves of illness, future pandemics or other significant disruptions to medical education (e.g. natural disasters), NPT could be a most useful format in fulfilling the learning needs of medical students in other programs.

## Tip 11: Adversity leads to innovation. Can we be innovative without relying on adversity?

The pandemic has led many medical programs to innovate - moving tutorials online, creating methods of assessment not reliant on face-to-face interaction and radically redesigning what elements of the course were crucial in order to ensure competent health professionals.

Although these innovations were adaptations to a crisis, the eventual resolution of such adversity need not mean that these improvements and flexibilities should be lost. While we may soon be in a period where adversity is no longer at the scale of an unprecedented pandemic, providers will always have a notable proportion of their students who are facing greater-than-average barriers to completing their degree.

Greater flexibility in engaging with content, attending placements and assessments opens up the study of medicine to a greater range of students. It creates opportunities for rural students, parents, students with disability, and students from disadvantaged backgrounds who may struggle fitting rigid timetables around working to support themselves.

As the COVID-19 situation has improved in Australia, and most states now head towards a more ‘normal’ landscape, educators should not simply look to return to ‘business as usual’ but consider whether current innovations can be maintained to empower students to overcome adversities in their daily lives. Many universities have shown creativity in being reactive to the challenges COVID-19 presented them, but we now have a chance to use that same creativity proactively, to push forward and tackle problems that have faced medical education for decades.

Many students have had a year so disrupted that they have no expectation of their academic experience fully returning to the original blueprint. We are hence at a unique, watershed moment where students and other stakeholders in medical education are better positioned to champion fresh and innovative approaches than ever before.

If it was possible to adapt curricula to meet the challenge of a pandemic, then why not redesign them to allow students to complete larger portions of their degrees in rural areas with the aim of tackling the rural workforce crisis? Could this not be the moment to shake up assessment mechanisms, trialling models which favour competency and clinical experience rather than ranking students and creating competitiveness at the expense of mental health and student co-operation? The adversity generated by COVID-19 has us temporarily seated in a once-in-a-generation window to introduce new solutions to old problems. It would be foolish to let this opportunity slip.

## Tip 12: Find the silver linings - and keep them

Clearly, the COVID-19 pandemic has brought about overwhelming and largely unquantifiable devastation across social, health and economic spheres worldwide. While 2020 has been a year underscored by significant loss, a reflective glance back reveals some silver linings. Though perhaps counterintuitive, distance and time apart were not always at the expense of connectedness. In many ways, the COVID-19 crisis catalysed a formidable camaraderie both within and between student and educator groups, one which bridged physical separation.

For example, one university described enhanced cross-site communication between senior students on rural placements. Many students, especially those on solo in remote placements, organised weekly group calls with each other as an opportunity for additional human contact. Ironically, this initiative would have been just as constructive and readily available before the pandemic, though it was the perception of additional, pervasive isolation this year that likely motivated it. More generally, the need for rapid, bilateral communication and troubleshooting between faculty, students, and other stakeholders broke down administrative walls and saw more of a unified front in medical education than ever before. Is this not the type of partnership we should strive for under normal circumstances as well? On a global scale, even those most affected by COVID-19 have begun to uncover silver linings of their own. A research team based in Wuhan, China, the original epicentre of the pandemic, recently published findings of a significant reduction in most air pollutants during the lockdown period ( Lian *et al*., 2020). Similar environmental benefits have been evidenced in the anomalously clear lagoon water of Venice, Italy ( Braga *et al*., 2020).

Ultimately, it appears that COVID-19 has functioned as a ‘common enemy’ and, in doing so, unified many formerly disparate groups. Indeed, the very authors of this article largely became acquainted owing to a national panel they hosted regarding the pandemic’s impact on medical students! As we now look towards medical education in the post-COVID era, may we remember and retain the hidden gems unearthed by the disruption.

## Conclusion

Mike Leavitt, the Secretary of the U.S. Department of Health and Human Services observed in 2007, ‘Everything we do before a pandemic will seem alarmist. Everything we do after a pandemic will seem inadequate.’ ( Leavitt, 2007)

We have likely all found some treasured insights while navigating the treacherous waters of the COVID-19 crisis. These *Tips* seek to impart the fundamental lessons we personally gleaned from the pandemic, and we trust that medical educators across a range of settings may find them of use. While there is much more yet to emerge we are encouraged by the lessons learned thus far, which we hope provides a sturdy blueprint moving forward. In brief, regular, bilateral communication will always be appreciated by students. The same can be said for co-designing curriculum changes and developing strong partnerships with student leadership. Practising kindness is a useful rule not only in medical education but also in life. We understand clearly the need to empathise with individuals. Students have taught us about skilful networking, and we have learned firsthand the depth and value of student channels. Across all facets of our lives in 2020, we have needed to define what is truly essential and the same can be said for medical curricula. We have learned about creative utilisation of Information Technology. Meaningful student participation in placements assumed a grim relevance in the pandemic and should not be forgotten. So, too, should we continue to promote near peer teaching and support. We have developed and delivered remarkable innovation from adversity (including tech-enhanced learning) that should not be lost in times of smoother sailing, and have found many silver linings - such as these *Tips!*

Much of the COVID-19 response has been about effective collaboration and communication. Let us carry this mindset into the future and ensure educators continue to work with, listen to and learn from medical students.

We are all constantly learning. However, as one webinar participant from Bond University noted, ‘this has been a tutorial like no other.’

## Take Home Messages

• Challenges associated with the COVID-19 pandemic have forced many changes upon medical education worldwide, not all of which have been detrimental
• Working collectively to tackle these challenges has brought students and educators closer together and led to a wide range of innovations
• 2020 has presented us with many lessons for the future that we can and should carry forward

## Notes On Contributors

**Julian Pecora** is a recent medical graduate from James Cook University and President of the James Cook University Medical Students’ Association.

**Tarun Sen Gupta** is Professor of Health Professional Education at the James Cook University College of Medicine and Dentistry. ORCiD: https://orcid.org/0000-0001-7698-1413

**Daniel Zou** is a medical student at the University of Melbourne and President of the Australian Medical Students’ Association. ORCiD: https://orcid.org/0000-0002-7320-4683

**Kathy Kwan** is a medical student at The University of Queensland and Chair of the International Sub-Committee of the University of Queensland Medical Society.

**Hannah Matthews** is a recent medical graduate from the University of Western Australia and Chair of the Medical Students’ Council of Western Australia.

**Steve Trumble** is Professor and Head of Medical Education at the University of Melbourne. ORCiD: https://orcid.org/0000-0002-8097-5785
