# Supplementary material for: Twelve things we have learned from medical students during the Pandemic
Source: MedEdPublish (2016). 2021 Mar 25;10:77. [Version 1] doi: 10.15694/mep.2021.000077.1 (PMC10939641; doi:10.15694/mep.2021.000077.1)
Supplement: Peer review history

# Reviewer report: Reviewer response for version 1

**DOI:** 10.21956/mep.18960.r27143

**Published:** 2021-06-14

**Authors:** Ken Masters

**Affiliations:** 1 Sultan Qaboos University

This review has been migrated. The reviewer awarded 4 stars out of 5 This is an interesting 12-tips paper based on Australian medical students’ experiences with online teaching and learning during Covid-19.Unlike many 12-tips papers that are focused on merely delivering online education, this paper is a great deal broader and takes into account a wide range of influencing factors. Although some of the information is local only, most is more widely applicable, and, even where local examples are used, readers will be able to see where their own environment relates to the issues.Probably my only criticism of the paper is that the Abstract should have summarised the main points. (Another reviewer has also noted the weakness of the rather broad statement: “Students are experts in the curriculum, and therefore ideally placed to advise on changes”) Other than that, a fine and useful read.

Reviewer Expertise:

NA

No decision status is available

# Reviewer report: Reviewer response for version 1

**DOI:** 10.21956/mep.18960.r27142

**Published:** 2021-05-04

**Authors:** Balakrishnan Nair

**Affiliations:** 1 Centre for Medical Professional Development and University of Newcastle

This review has been migrated. The reviewer awarded 5 stars out of 5 I enjoyed reading this well written article . It is good to see students taking a lead in educational research and publication, since they are the future of our profession. As we all know it is all about learning and NOT about teaching. It took a pandemic for us to learn these tips! I believe all medical educators can learn from the voice of the learners and these tips are very valuable . I particularly liked the dictum to 'translate compassion into pedagogy' . In medicine we need to shift our focus from asking what is the matter with you to what matters to you ! If we can ask that to our patients, we should ask the same question to the learners too .

Reviewer Expertise:

NA

No decision status is available

# Reviewer report: Reviewer response for version 1

**DOI:** 10.21956/mep.18960.r27141

**Published:** 2021-04-10

**Authors:** Rajaei Sharma

**Affiliations:** 1 College of Medicine and Health, University of Exeter

This review has been migrated. The reviewer awarded 4 stars out of 5 This is a very nicely written piece with important lessons learned from a vast cohort of students. It is likely to be of continued value to medical educators, especially those that are programme leads and developers. Addressing the minute issues around clarity raised below would likely vastly increase the reach of this paper and its readership. The title is (perhaps intentionally?) a little vague which can be appropriate at times but must be contrasted with a particularly engaging and explicit abstract which is lacking here. The abstract, although well written, reads as an introduction and unfortunately the reader is none the wiser as to the actual content of the paper. The introduction is well written with a pleasing depth to the background, especially as this journal has a global readership. It is well structured and leads the reader on the journey. The only factor lacking in the introduction (and the abstract) is a pointed statement as to who the piece is targeting; it’s only once we are into the tips themselves that we quickly see it’s for the educators and not particularly relevant for students. The tips are well written and again have an appreciable depth. Some tip headers (4/5/8) can be shortened and/or made more pertinent. This will then act as a sharp primer for the text ahead but also mean that the reader can easily revisit a particular section quickly without having to try to figure out where it might be. The concluding paragraph of tip 5 is especially useful and could perhaps be repeated with some of the other longer/more prescriptive tips to enhance clarity.Some of the tips are much more prescriptive than others. This is absolutely relevant in this context, but it may be additive to have a more pointed recall to these in either the conclusion or take-home messages. Each and every tip is useful but highlighting (and so reinforcing) the most valuable adaptations may be beneficial. Overall, a great piece that is highly applicable to the readership of MedEdPublish and the wider educational community!

Reviewer Expertise:

NA

No decision status is available

# Reviewer report: Reviewer response for version 1

**DOI:** 10.21956/mep.18960.r27140

**Published:** 2021-04-01

**Authors:** Michelle McLean

**Affiliations:** 1 Bond University

This review has been migrated. The reviewer awarded 5 stars out of 5 Loved reading this paper. At the same time as reviewing, I was engaged in a Teams discussion about the value of online learning. Some were arguing for F2F still being an important part of learning. The article is extremely well written and is jam packed with useful strategies. The strength is that is written from a student perspective and highlights the value of the staff-student partnership in terms of curriculum which epitomises learner-centredness. All educators should read this article to gain an insight of what it was like to be students during COVID. Tip 4 was my favourite. A small reservation with this statement: "Students are experts in the curriculum, and therefore ideally placed to advise on changes". This is a sweeping statement. While I agree that senior students who have experienced the curriculum in action, including the hidden curriculum, could be considered 'experts' in terms of the curriculum that they have experienced, students who enter as 17 or 18 year olds, are largely naive not only in terms of their life experiences but also in terms of their previous educational background. In addition, most students have not undertaken any educational studies to understand the underpinning theories relating to learning and teaching and assessment. Many academics have professionalised their practice by becoming educators (rather than teachers) through post-graduate certification or degrees in health professional or medical education and so are educational scholars. Hopefully, what and how they do what they do is informed by evidence. The gap, however, is often in technology. Our current students are far more digitally literate than most academics and so pivoting to remote 'teaching' was a very steep learning curve for many and in a very short time. For some academics, this caused much distress. Notwithstanding, with help from colleagues, Offices of L&T and from students, we managed to deliver a whole semester in most instances entirely remotely. Congratulations on a great article!

Reviewer Expertise:

NA

No decision status is available
